# Supplementary material for: Artificial Feeding of Ornithodoros fonsecai and O. brasiliensis (Acari: Argasidae) and Investigation of the Transstadial Perpetuation of Anaplasma marginale
Source: Microorganisms. 2023 Jun 28;11(7):1680. doi: 10.3390/microorganisms11071680 (PMC10385771; doi:10.3390/microorganisms11071680)
Supplement: Supplementary file 1 [file microorganisms-11-01680-s001.zip › microorganisms-2422251-supplementary.pdf]

Table S1 – Primer sequences according to the target genes used in PCR assays.

<table>
<tr><th>Agents</th><th>PCR type</th><th>Target gene</th><th>Nucleotide sequence (5'-3')</th><th>Reference</th></tr>
<tr><td rowspan="2">Mammal</td><td rowspan="2">cPCR</td><td rowspan="2">gapdh</td><td>GAPDH-F: CCTTCATTGACCTCAACTACAT</td><td rowspan="2">23</td></tr>
<tr><td>GAPDH-R: CCAAAGTTGTCATGGATGACC</td></tr>
<tr><td rowspan="3">Anaplasma marginale</td><td rowspan="3">qPCR</td><td rowspan="3">msp1β</td><td>AM-F: TTGGCAAGGCAGCAGCTT</td><td rowspan="3">24</td></tr>
<tr><td>AM-R: TTCCGCGAGCATGTTGCAT</td></tr>
<tr><td>Probe: 6FAM-TCGGTCTAACATCTCCAGGCTTTCAT-BHQ1</td></tr>
<tr><td rowspan="2">Tick</td><td rowspan="2">cPCR</td><td rowspan="2">16S rRNA</td><td>16S-1: CCGGTCTGAACTCAGATCAAGT</td><td rowspan="2">25</td></tr>
<tr><td>16S+1: CTGCTCAATGATTTTTTAAATTGCTGTGG</td></tr>
<tr><td rowspan="3">Anaplasma marginale</td><td rowspan="3">snPCR</td><td rowspan="3">msp1α</td><td>F1: GTGCTTATGGCAGACATTTCC</td><td rowspan="3">26</td></tr>
<tr><td>Re: CTCAACACTCGCAACCTTGG (1st and 2nd rounds)</td></tr>
<tr><td>msp1αNF: CGCATTACACGTTCCGTATG</td></tr>
</table>
